# Quasi-Static Multifunctional Characterization of 3D-Printed Carbon Fiber Composites for Compressive-Electrical Properties

**DOI:** 10.3390/polym14020328

**Published:** 2022-01-14

**Authors:** Ritesh Ghimire, Frank Liou

**Affiliations:** Department of Mechanical and Aerospace Engineering, Missouri University of Science and Technology, Rolla, MO 65409, USA; liou@mst.edu

**Keywords:** quasi-static, compressive strength, multifunctional characterization, 3D printing, additive manufacturing, carbon fiber composites, Compressive-Electrical properties

## Abstract

Multifunctional carbon fiber composites provide promising results such as high strength-to-weight ratio, thermal and electrical conductivity, high-intensity radiated field, etc. for aerospace applications. Tailoring the electrical and structural properties of 3D-printed composites is the critical step for multifunctional performance. This paper presents a novel method for evaluating the effects of the coating material system on the continuous carbon fiber strand on the multifunctional properties of 3D-printed composites and the material’s microstructure. A new method was proposed for the quasi-static characterization of the Compressive-Electrical properties on the additively manufactured continuous carbon fiber solid laminate composites. In this paper, compressive and electrical conductivity tests were simultaneously conducted on the 3D-printed test coupons at ambient temperature. This new method modified the existing method of addressing monofunctional carbon fiber composites by combining the monofunctionality of two or more material systems to achieve the multifunctional performance on the same component, thereby reducing the significant weight. The quasi-static multifunctional properties reported a maximum compressive load of 4370 N, ultimate compressive strength of 136 MPa, and 61.2 G Ohms of electrical resistance. The presented method will significantly reduce weight and potentially replace the bulky electrical wires in spacecraft, unmanned aircraft systems (UAS), and aircraft.

## 1. Introduction

Quasi-static monofunctional structural testing was conducted to characterize the tensile and fatigue properties of the 3D-printed polylactic acid (PLA)–graphene and the effects of the process parameters on the strength and fatigue behavior of the test specimens [1]. While this paper compiled useful data about the effects of process parameters on static and fatigue behavior, and tensile strength and fatigue life of the 3D-printed PLA–graphene specimens, it did not address the quasi-static multifunctional characterization of the 3D-printed carbon fiber composites for Compressive-Electrical properties [1]. In fact, it evaluates the effect of process parameters based on the nature of this process, which is classified as a thermally driven process [1].

Monofunctional electrical properties of the 3D-printed continuous carbon fiber composites fabricated by using the Markforged MarkTwo^®^ fused deposition modeling (FDM) were investigated [2]. While the electrical conductivity tests of the composites revealed promising results, this paper did not include the multifunctional quasi-static characterization of the 3D-printed continuous carbon fiber slid laminates for Compressive-Electrical properties [2]. An alternative material to polymer sizing, called carbon nanotubes (CNTs) was introduced on the carbon fibers to improve electrical and thermal functionalities [3]. The CNT-modified CFRP showed remarkable electrical conductivity improvements in all three directions, with significant enhancements in the surface, thickness, and volume [3]. While the technology seemed promising, the scalability and CNT fabrication methods and processes were not practical due to inherent drawbacks; for example, it is a tedious and time-consuming process that damages carbon fibers. Compressive strength [4] characterizing polyamide 6 reinforced with carbon fiber specimens was conducted using AM technology based on composite filament fabrication (CFF*) [5]. In this method, the CFF* utilized a similar layer-by-layer printing, as fused filament fabrication (FFF), but it also reinforced parts with layers of various continuous fibers into the polymer matrix [5]. While this study conducted a detailed investigation on compressive and flexural strengths of 3D-printed coupons, multifunctional continuous carbon fiber composites were not addressed. A traditional layup method was used to manufacture a multifunctional composite battery that included lithium-ion battery active materials with carbon fiber weave materials to form energy-harvesting carbon fiber composites [6]. Structural composite battery panels were found to be integrated power harvesting platforms for the 1U CubeSat frame to supplement or replace interior and external battery packs [6]. The effects of compression loading on composite laminates’ mechanical and electrical responses were examined. The installed batteries showed adverse effects on the compressive stiffness, failure stress, and laminates’ fatigue life [7]. The minimization of the properties’ impact was not addressed, which may be achieved using optimized placement and orientation of the batteries [7].

The 3D printing (3DP) method, including light-based 3DP and ink-based 3DP, is a rapidly developing technology, which has received much attention of late [8]. The raw materials for the light-based 3DP are limited even though this AM technology provides higher feature resolution [8]. On the other hand, the raw materials for ink-based 3DP are widely available and more compatible with various materials, thus providing wide applications [8]. Graphene-based materials have been extensively investigated in ink-based 3DP owing to their unique properties such as high conductivity and superior mechanical flexibility [8]. The ink-based 3DP of graphene-based raw materials, their basic properties, and preparation methods were reviewed in [8]. Different types of ink-based 3DP such as FDM, direct-write assembly, and inkjet printing technology were also reviewed in detail, with special emphasis on 3D printing methods of graphene-based materials, as-printed architecture’s performance, and their uses [8]. While the detailed review was presented, this paper did not address the novel quasi-static multifunctional characterization of the additively manufactured multifunctional continuous carbon fiber composites for Compressive-Electrical multifunctional properties [8]. Thermosets are widely used raw materials for 3D printing for aerospace applications due to their remarkable specific strength, thermal stability, and chemical resistance [9]. Manufacturing and processing of composites irrespective of thermoplastic or thermosets are always challenging, and of course, using the AM technique also adds more complications [9]. While a detailed review was presented regarding the AM of thermosets, this paper did not address the multifunctional characterization of the 3D-printed multifunctional continuous carbon composites for Compressive-Electrical properties [9]. While laser sintering of polymers and the limited validity of the model of isothermal laser sintering were shown by experiments, this paper did not address the quasi-static characterization of the additively manufactured multifunctional continuous composites for Compressive-Electrical properties [10]. Advances in AM of thermoplastic polymer composites and nanocomposites with respect to the importance of the thermoplastic categorization into particle-, fiber-, and nanomaterial-based composites and polymer blends are well addressed [11]. While the FDM of thermoplastics and the different types of the AM techniques that allow higher filler loading for the thermoplastics such as liquid deposition modeling, also known as direct ink writing, are discussed in detail [11], this paper did not address the multifunctional characterization of continuous carbon composites for multifunctional Compressive-Electrical properties [11]. While the multimaterial stereolithography AM device was developed for the fabrication of parts in the micrometer range, this paper did not address the quasi-static multifunctional characterization of the continuous carbon fiber composites for Compressive-Electrical properties [12]. Volumetric AM via tomographic reconstruction technique for multi-material fabrication was presented in which concurrent printing of all points within a 3D object by illuminating a rotating volume of photosensitive material with a dynamically evolving light pattern [13]. This paper did not address the multifunctional characterization of the multifunctional carbon composites for Compressive-Electrical properties [13]. Implosion fabrication was developed that involved the direct assembly of 3D nanomaterials made up of metals, semiconductors, and biomolecules arranged in 3D geometry in which hydrogels were presented as scaffolds for volumetric deposition of materials at defined points in space [14]. These scaffolds were optically patterned in 3D, installed in one or more functional materials, and then shrank and dehydrated in a controlled way to obtain nanoscale feature sizes in a solid substrate [14]. While the implosion fabrication was developed to write conductive 3D silver nanostructures within an acrylic scaffold via volumetric silver deposition in the tens of nanometers resolutions for optical applications, this paper did not address multifunctional Compressive-Electrical properties of 3D-printed carbon composites [14]. Two-photon lithography (TPL)-based submicrometer AM was used for spatially and temporally focusing an ultrafast laser to implement a projection-based layer-by-layer parallelization to increase the output and geometric design space [15]. This paper did not address the multifunctional Compressive-Electrical properties of 3D-printed carbon composites [15]. The tensile properties of diverse concentric fiber rings and fiber layers were investigated to characterize the 3D-printed composites [16]. While the increase in concentric fiber rings and fiber layers is attributed to increase in tensile strength and modulus, this paper did not address the multifunctional Compressive-Electrical properties of the continuous carbon fiber composites [16]. Thermal studies of the AM using pulsed laser heating was investigated in which, a multiphysics simulation was conducted for predicting temperature rise and curing profile of polydimethylsiloxane (PDMS) heated by a periodic pulsed laser [17]. While the simulation involved coupling of local heating and curing change, this paper did not address the multifunctional characterization of multifunctional carbon composites for Compressive-Electrical properties [17].

The compressive properties of continuous carbon fiber-reinforced thermoplastic were investigated by testing composite specimens that were additively manufactured using the FFF [18]. The results suggested that the thermoplastic resin’s composition was different for the unreinforced and reinforced filaments [18]. Compression testing was conducted on additively manufactured short-fiber-reinforced thermoset composites test coupons to measure the mechanical performance [19]. Milled carbon fibers were used as the reinforcing fibers, which were considered too short to enhance the mechanical strength of composites [19]. The AM process was used to manufacture continuous and long fiber-reinforced composite parts using a 3D printer to assess the compressive properties [20]. The mechanical properties of AM composites were not comparable to traditional methods due to high porosity [20]. Experimental investigations for compressive properties of 3D-printed functional prototypes fabricated using fused deposition modeling were conducted [21]. Non-additively manufactured graphene nanoplatelets were used to investigate the compression experiments with simultaneous electrical measurements for electromechanical response [22]. Out-of-plane electrical conductivity in traditionally manufactured polymer composites was enhanced by using a CO_2_ laser as a means of nanostructuring the surface of carbon fiber (CF) tows in an incessant throughput procedure [23]. The results demonstrated an increase in out-of-plane electrical conductivity, while it preserved Mode-I interlaminar fracture toughness of the laminate composite, showing multifunctionality potential [23].

Monofunctional analyses were carried out of aircraft structural components made up of additively manufactured composites that were matured. The electrical conductivity in the composites depends on the amount and orientation of carbon fibers present. A significant amount of research was conducted on the monofunctional properties of the advanced carbon composites, which enhanced the maturity of the carbon composites’ multifunctional performance. Monofunctional electrical and strain sensing mechanisms (electromechanical properties) of hybrid graphene nanoplatelet (GNP)/carbon nanotube (CNT)-reinforced composites were investigated [24]. While the test results showed good inferences, this study did not include the experimentation of the 3D-printed specimens for the multifunctional Compressive-Electrical properties [24].

This research examined coupled multifunctional compressive and electrical characterization and the development of additively manufactured continuous carbon fiber composites. Hence, in this work, simultaneous compression and electrical conductivity tests were executed on the continuous carbon fiber test coupons.

## 2. Materials and Methods

### 2.1. Materials for 3D Printing of Coupons

**Figure 1:**
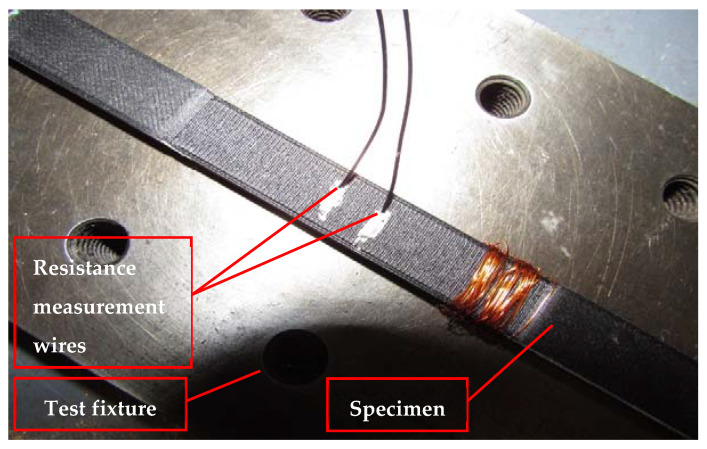
Test specimen with instrumentation for measurement of the electrical resistance of the test specimens.

The multifunctional continuous carbon fiber solid laminate composites were designed by selecting the raw material manufactured by the Markforged Company (Watertown, MA, USA). Onyx with continuous carbon fiber supplied by Markforged was used [25]. The Onyx FR is a UL 94 V-0 Blue Card certified down to a thickness of 3 mm [26]. The carbon fiber was selected based on pre-defined criteria that required it to be longitudinally strong and commonly used in aerospace applications. A high strength-to-weight ratio was important in this design and selection. Markforged X7 AM machine (Markforged Company, Watertown, MA, USA) was used for the test coupons fabrication. The 3D-printed continuous carbon fiber solid laminate coupons were manufactured using the particular type of the FFF AM method called the continuous filament fabrication (CFF) for this work [27]. The use of the CFF [28] requires an additional nozzle to lay the additional material on the 3D-printed test coupons. Markforged X7 model’s Industrial Composite 3D Printers series was used at RE3DTECH company [29] located in Grayslake, IL, USA, with a raster angle of 0 degrees for the specimen fabrication. The fiber layout was in line with the longest dimension of the test coupons. The temperatures of the Onyx printing and fiber-laying nozzles were 275 °C and 250 °C, respectively. The diameters of the extruded Onyx and fiber materials were 0.40 mm and 0.9 mm wall thickness, respectively. The surface finish on the 3D-printed coupons was 0.125 mm layer height. The specimens were 3D-printed flatwise, and the continuous carbon fiber was laid zero degrees along the longitudinal axis of the specimens. The test specimens with instrumentation for measurement of the electrical property of the test specimens are shown in Figure 1.

### 2.2. Methods for Multifunctional Characterization

**Figure 2:**
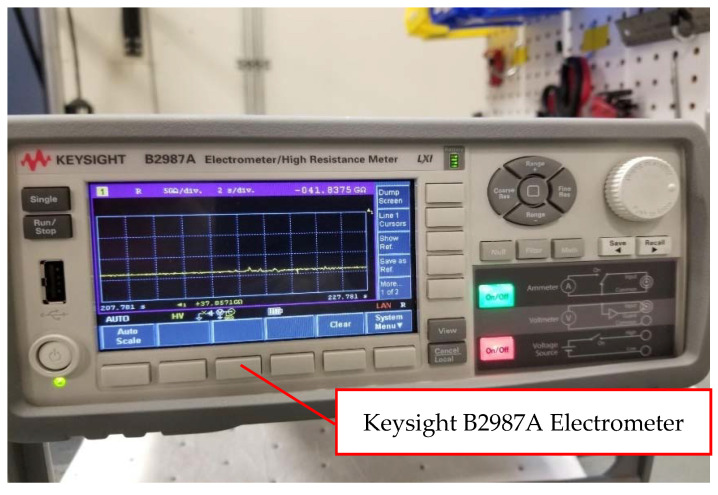
Keysight B2987A Electrometer (Reproduced with Permission, Courtesy of Keysight Technologies, © Keysight Technologies, Inc., Santa Rosa, CA, USA) [34] for measurement of the electrical property of the test specimens.

**Figure 3:**
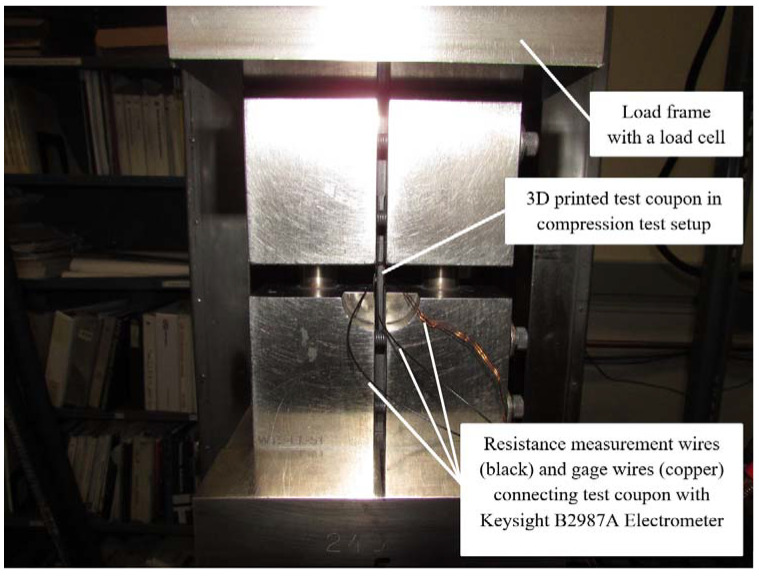
Multifunctional Compressive-Electrical characterization test set up on MTS machine at RTD.

Silver paint, wire, and conductive epoxy glue were used to attach wires to the specimens [23,30,31,32,33]. The distance between the two electrical contacts was reported for each specimen. Evaluations of the compressive and electrical properties were conducted simultaneously, while the AM composites test coupons underwent electrical conductivity and flexural tests. Test coupons were connected with a Keysight B2987A electrometer [34] for measuring electrical conductivity from the ends along the test coupons’ lengths. The compression test used for this research was conducted per ASTM D6641 [35]. Keysight B2987A electrometer employed to measure the electrical resistance is shown in Figure 2. Quasi-static multifunctional Compressive-Electrical tests were conducted at a constant rate of 1.27 mm/minute on the MTS machine [36]. The testing temperature was 72.5 ± 7.5 °F, and the relative humidity for the testing was ambient. The multifunctional Compressive-Electrical characterization test set up on the MTS machine at room temperature dry (RTD) is shown in Figure 3. National Institute for Aviation Research (NIAR) [37] AGATE-WP3.3-033051-102 defined RTD was chosen for this investigation [38]. The average dimensions of the additively manufactured test specimen’s length × width × thickness were 139.5 mm × 12.6 mm × 2.6 mm, and the average distance between the electrical contacts on the test coupons was 8.1 mm. The test coupons were loaded in intervals to best approximate the static behavior of the test coupon material for measuring electrical resistance. The measurements were allowed up to 10 min to settle. Detailed explanation and discussion about the settling time are included in the Results and Discussion Section. A strain gage was attached to the gage area of one side of the test specimen, and the resistance measurement was measured on the opposite side of the test specimen. The resistance measurement wires were attached to the test specimens using a special high temperature and moisture-resistant adhesive. Figure 3 shows the multifunctional Compressive-Electrical characterization test setup of 3D-printed specimens on the MTS machine at RTD. The resistance measurements were taken every 334 N until the failure of the experimented test specimens. A few test specimens were monofunctionally tested on the MTS machine to evaluate the structural failure loads of those test specimens. Based on the reported failure loads of the monofunctionally tested specimens for structural properties (compressive strength and load), the number of the intervals were evaluated by choosing 334 N as the interval load for this research investigation to access the multifunctional Compressive-Electrical properties of the 3D-printed carbon fiber composites test specimens. The coupled multifunctional compressive and electrical characterization and development of additively manufactured continuous carbon fiber composites tests were conducted in this research. The results were revealed and discussed in detail in the Results and Discussion Section of this paper.

## 3. Results and Discussion

**Figure 4:**
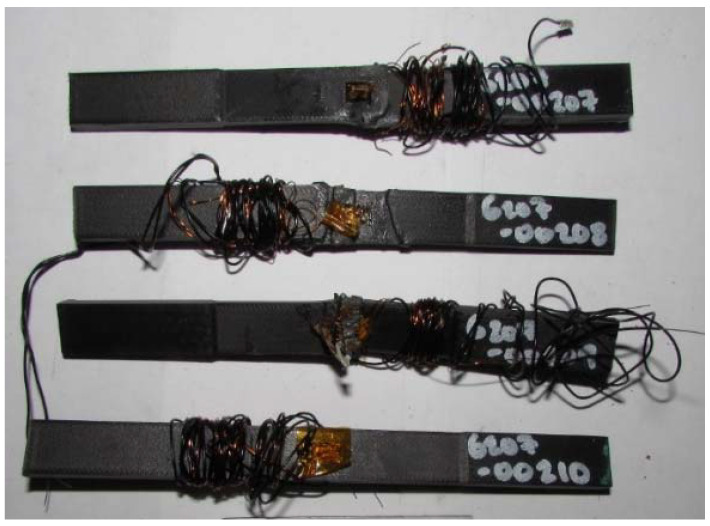
Failed test coupons after the RTD multifunctional Compressive-Electrical test.

**Figure 5:**
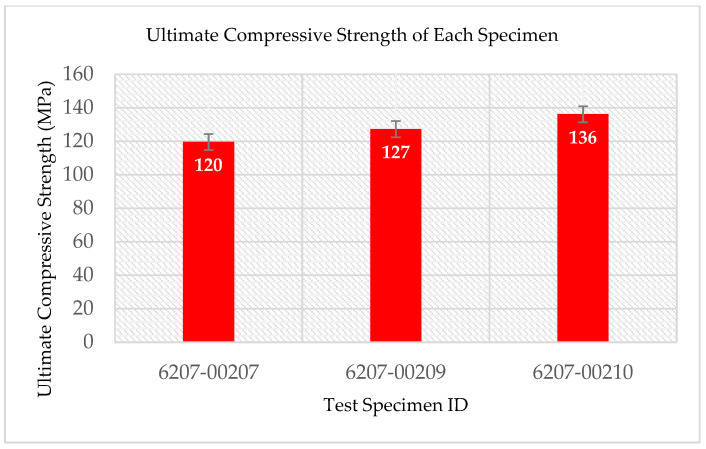
Ultimate compressive strength of each specimen after the RTD multifunctional Compressive-Electrical test.

**Figure 6:**
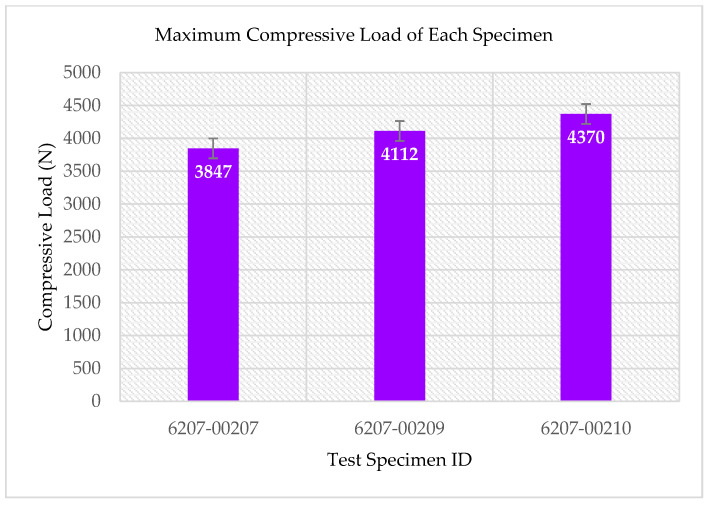
Maximum compressive load of each specimen after the RTD multifunctional Compressive-Electrical test.

**Figure 7:**
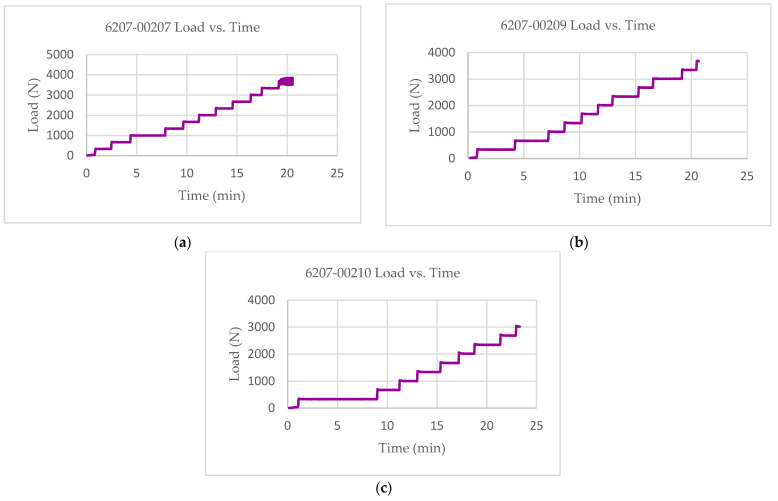
Load–time behavior of multifunctional test coupons at RTD: (**a**) 6207-00207; (**b**) 6207-00209; (**c**) 6207-00210.

**Figure 8:**
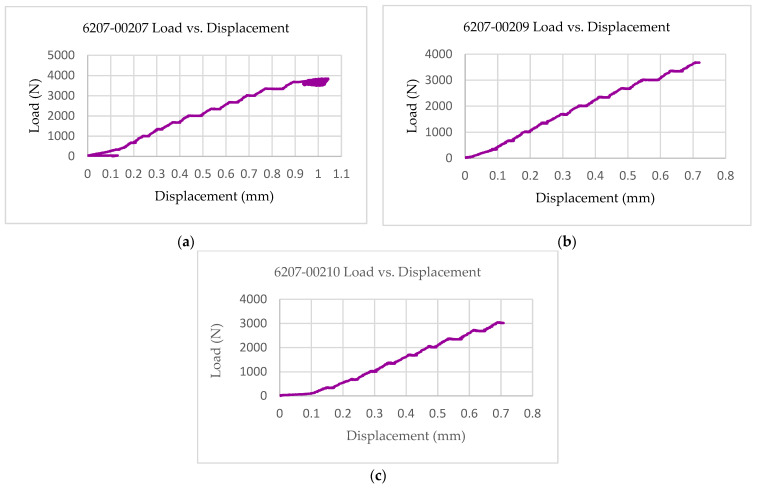
Load–displacement behavior of multifunctional test coupons at RTD: (**a**) 6207-00207; (**b**) 6207-00209; (**c**) 6207-00210.

**Figure 9:**
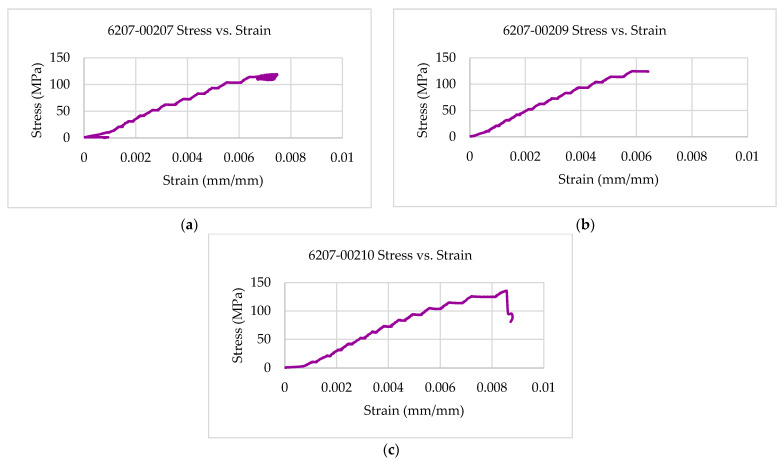
Strain–stress behavior of multifunctional test coupons at RTD: (**a**) 6207-00207; (**b**) 6207-00209; (**c**) 6207-00210.

The multifunctional quasi-static Compressive-Electrical characterization of additively manufactured continuous carbon fiber composites test coupons was conducted using Keysight B2987A Electrometer and MTS testing machine. Figure 4 shows the failure modes of tested coupons after the RTD multifunctional quasi-static Compressive-Electrical test. The test articles exhibited a maximum compressive load of 4370 N, a corresponding compressive strength of 136 MPa, and 61.2 G Ohms of resistance as multifunctional properties. The ultimate compressive strength of each specimen after the RTD quasi-static multifunctional Compressive-Electrical test is depicted in Figure 5. Similarly, the maximum compressive load of each test coupon after the RTD multifunctional Compressive-Electrical test is presented in Figure 6. Figure 7 shows the loading versus time behavior of the 3D-printed multifunctional test coupons at RTD. Figure 8 presents the load versus displacement behavior of the test coupons at RTD. As shown in Figure 7 and Figure 8, the stepwise looking behavior of the tested multifunctional continuous carbon fiber solid laminate composites test specimens during the multifunctional Compressive-Electrical testing was contributed by the presence of noise level that required some time to settle and achieve a stable plot on the electrometer. Figure 9 shows the stress versus strain behavior of the multifunctional test coupons. It is worth noting that the stress recorded in the plots are nominal values.

**Figure 10:**
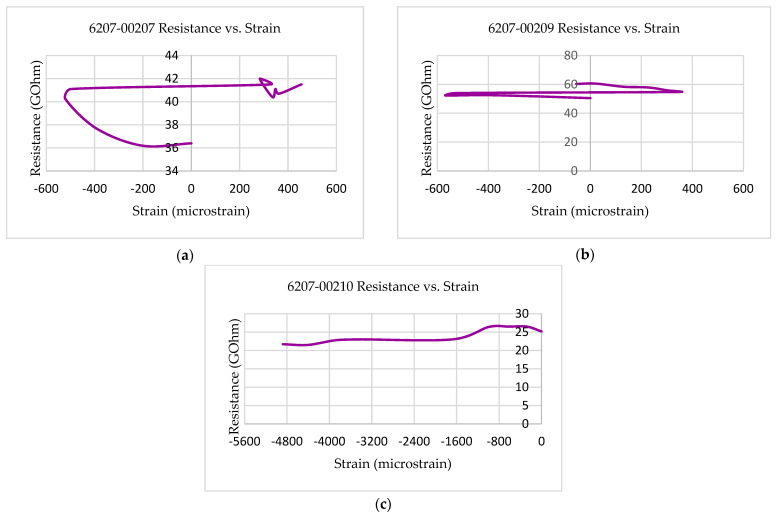
Multifunctional compressive-electrical properties of test coupons at RTD: (**a**) 6207-00207; (**b**) 6207-00209; (**c**) 6207-00210.

Figure 10 shows the quasi-static multifunctional Compressive-Electrical properties of the 3D-printed multifunctional continuous carbon fiber test specimens at RTD in the form of resistance versus strain behavior. Figure 10 shows the strain values corresponding to the maximum resistance values for each of the test coupons (6207-00303, 6207-00304, and 6207-00305) that were observed to be lower than the failure strain of those test coupons. The strain at peak resistance value, when compared with the respective failure strain, was found to be 96.69% lower than the associated failure strains for the specimen (6207-00209). The strain corresponding to the maximum resistance value for specimen 6207-00207 was observed to be 37.76% lower than the associated failure strain for that specimen (6207-00207). Similarly, the strain corresponding to the associated failure strains for the specimen 6207-00210 was found to be 87.45% lower than the associated failure strain for that specimen (6207-00210). The experimental results showed that the resistance values of the multifunctional specimens during the multifunctional tests were observed to be higher than the residual resistance values for the specimens—6207-00207, 6207-00209, and 6207-00210. The residual resistance value (50.5 G Ohms) for specimen 6207-00209 was observed to be lower than the resistance values recorded during the tests (61.2 G Ohms, 17.48% higher than 50.5 G Ohms). The residual resistance value (25.2 G Ohms) for the specimen 6207-00210 was found to be lower than the resistance values recorded during the tests (26.5 G Ohms, 4.91% higher than 21.7 G Ohms). Similarly, the residual resistance value (36.4 G Ohms) for the specimen 6207-00207 was found to be lower than the resistance values recorded during the tests (42.0 G Ohm, 13.33% higher than 36.4 G Ohms).

**Figure 11:**
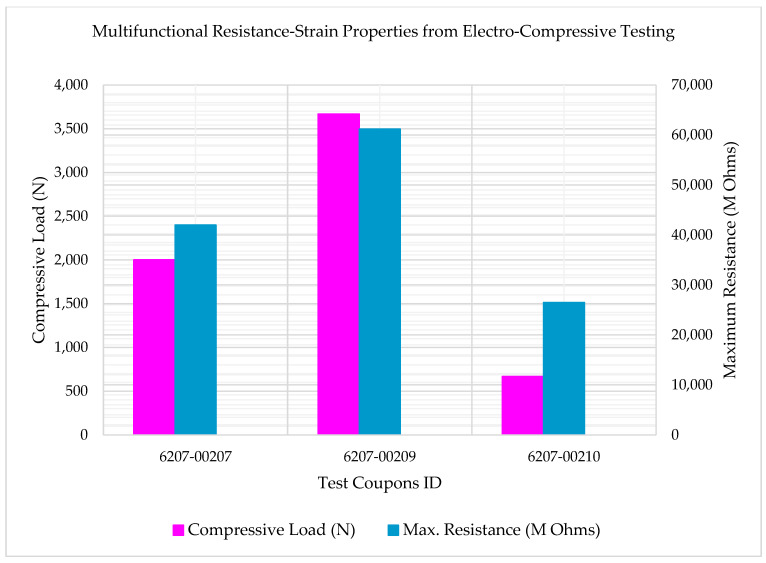
Multifunctional Compressive-Electrical behavior of test coupons (6207-00207, 6207-00209, and 6207-00210) at RTD.

Figure 11 shows the multifunctional Compressive-Electrical behavior of the test coupons (6207-00207, 6207-00209, and 6207-00210) at RTD and shows coupling effects of compressive strength and electrical properties of the 3D-printed multifunctional carbon fiber composites. In Figure 11, the coupling effects of the compressive load corresponding to the maximum electrical resistance values of each test specimen are presented. As shown in Figure 11, the coupled Compressive-Electrical multifunctional properties of the test specimens—6207-00207 and 6207-00210 are found to be consistent with respect to the reported higher maximum resistance than their corresponding compressive loads, while for the test specimen 6207-00209, the coupled Compressive-Electrical multifunctional properties are found to be in a state that involved the reported maximum resistance to be lower than its corresponding compressive load. As shown in Figure 11, the compressive load corresponding to the maximum resistance value of 61.2 G Ohms for the specimen 6207-00209 was found to be lower than the failure compressive load recorded during the tests (4003.4 N, 8.33% higher than 3669.8 N). The compressive load corresponding to the maximum resistance value of 26.5 G Ohms for the specimen 6207-00210 was observed to be lower than the failure compressive load recorded during the tests (2668.9 N, 75% higher than 667.2 N). Similarly, the compressive load corresponding to the maximum resistance value of 42 G Ohms for the specimen 6207-00207 was observed to be lower than the failure compressive load recorded during the tests (3336.2 N, 40% higher than 2001.7 N). This may be attributed to material and manufacturing defects. The slight variation in values could be attributed to the lack of electrical shielding, as well as the difficulty of the MTS machine to add the electrical shielding system. Additional parameters contributing to the slight variation in the values are (1) compression testing fixture restricting enough access for the test personnel, (2) effects of special electrical wiring adhesion on the test specimens for this multifunctional properties’ evaluation, and (3) other manufacturing and processing defects, as well as environmental effects.

The summary of the experimental results of the test specimens is shown in Table 1 and Table 2. Table 1 presents the compressive load profile of the test specimens. Table 2 shows the maximum electrical resistance encountered during the multifunctional Compressive-Electrical testing of the 3D-printed multifunctional carbon fiber composite solid laminates. The average value of the maximum electrical resistance reported during the multifunctional Compressive-Electrical testing was 43.23 G Ohms. This study also yielded an average maximum compressive load of 4110 N and average compressive strength of 128 MPa on the tested solid laminate 3D-printed test coupons in the multifunctionality testing. The compressive strength (128 MPa) obtained from this study was compared with the compressive strength of the IM7/PEEK composites (161.2 and 229.5 MPa) fabricated using automated fiber placement, and it was found to be 20.6% lower than 161.2 MPa and 44.2% lower than 229.5 MPa [39]. The compressive strength of this study was compared against values reported in the literature and was found lower than the strength of a typical carbon fiber unitape laminate validated by industry and the NIAR [40]. This may be attributed to the matrix material, and it is recommended that the matrix material properties be further explored in future studies to assess adhesion strength and properties per the widely accepted industry practices such as NIAR, NCAMP, etc. Additionally, the compressive strength reported here was also compared with an additively manufactured material system data of the NCAMP and was found to be 130% higher than the compressive strength of the Stratasys Certified ULTEM™ 9085/Fortus 900mc [41]. Notably, this material data has been validated by industry and the NIAR per the NCAMP protocols. The primary reason for higher values than the compressive strength reported in CAM-RP-2018-013 Rev A MPDR ULTEM 9085 is that the study presented here includes continuous carbon fiber and the Stratasys Certified ULTEM™ 9085/Fortus 900mc does not. Some of the main reasons that lead to knockdown of the compressive strength are as follows: (i) compression testing/strength is resin/matrix dominant testing; (ii) resin fails before fiber failure in compression testing; (iii) tension testing is fiber dominant testing; when the resin starts failing in tension testing, the fiber still carries load taking all the way to the ultimate failure load; (iv) in compression testing, the matrix/resin is a binding agent and is also a structural component that holds the majority of the structural loading. When the resin starts to fail, the embedded fiber (even though not structurally damaged) cannot handle loads and thus crumbles as in a typical buckling condition. While consolidation methodologies and their results with respect to fiber volume fraction, void content, and strength (flexural and tensile) for the 3D-printed continuous reinforcement composites were reviewed thoroughly for monofunctional material and property, it is recommended that further study be conducted to address the consolidation, voiding and quality of the 3D-printed multifunctional continuous carbon fiber solid laminates, as well as in situ consolidation for multifunctional Compressive-Electrical properties [42]. It was found that the mechanical properties of the 3D-printed continuous fiber-reinforced plastics (CFRP) are influenced by the shortcomings in consolidation application, and thermoset matrices of the 3D-printed CFRP exhibit better mechanical properties than for the thermoplastic matrices [42]. The quasi-static multifunctional Compressive-Electrical characterization was investigated, and the development of additively manufactured continuous carbon fiber solid laminate composites was conducted in this study. The failed test coupons after the RTD quasi-static multifunctional Compressive-Electrical testing suggest that this study is consistent with the traditionally manufactured carbon fiber composites, compared with the failure modes of the carbon-fiber-reinforced polymer matrix laminates composites [43]. The mechanisms and modes of the compressive failure and the coupling effects of the electrical properties on the compressive failure modes and the mechanisms were found to influence the laminate thickness on these failure modes and failure mechanisms. Linear and non-linear deformations contributed to a constant change of electrical resistance with a change in strain (in/in). This multifunctionality property is attributed to the progressive onset and propagation of microcracks in the 3D-printed continuous carbon fiber solid laminate coupons. The quasi-static experimental test results suggest that the 3D-printed test specimens did not possess a significant amount of void or delamination due to well-aligned test data. This inference was confirmed by using the AM technique to fabricate the multifunctional continuous carbon fiber solid laminate composites and compared with the traditionally manufactured carbon composites. Thus, the quasi-static multifunctional Compressive-Electrical characterization and the development of 3D-printed continuous carbon fiber solid laminate composites are appropriate for aerospace applications.

## 4. Conclusions

Traditional and existing manufacturing technologies and methods for multifunctional carbon fiber composites are not well investigated. These technologies and multifunctional composites are not widely implemented on a large commercial scale due to a lack of research findings. This research characterized the integrated quasi-static multifunctional Compressive-Electrical performance and supported the development of additively manufactured continuous carbon fiber composites. In this study, simultaneous quasi-static compression and electrical conductivity tests were conducted on 3D-printed continuous carbon fiber test coupons for their suitability for aerospace applications. This investigation showed that the test coupons exhibited a quasi-static maximum compressive load of 4370 N, a corresponding ultimate compressive strength of 136 MPa, and 61.2 G Ohms of resistance as multifunctional properties. An average full compressive load of 4110 N and an average ultimate compressive strength of 128 MPa on the tested solid laminate 3D-printed test coupons were noted. The failure modes of the test coupons suggest that the results are consistent with the traditionally manufactured carbon fiber composites. A constant change of electrical resistance with respect to a change in strain (in/in) was contributed by deformations. This quasi-static multifunctionality property can be attributed to the onset and propagation of microcracks in the 3D-printed coupons. Material, processes, and environmental conditions’ variabilities appear to be negligible due to the AM of the test coupons and the exhibited experimental test results, suggesting that the 3D-printed coupons did not have void or delamination in them. Thus, the quasi-static multifunctional Compressive-Electrical characterization is appropriate for an aerospace application because this proposed methodology significantly reduces the overall weights of spacecraft, UAS, and aircraft. While the AM of monofunctional composites and their monofunctional properties’ characterization appear to be well explored, the quasi-static multifunctional characterization of 3D-printed multifunctional continuous carbon fiber solid laminates, especially, the coupled Compressive-Electrical properties are not addressed in the literature. This is the main highlight and novelty of this study, and the research findings presented here will significantly contribute to the literature and to the large engineering community and industry. The current work shows that there is a definite correlation between compressive strength and resistance of the multifunctional carbon fiber composites material system. Hence, this methodology can be extended to a structural health monitoring system, where the change in resistance can be correlated to a decrease in the strength of the composite. Coupled with advanced manufacturing methods, the multifunctional composites pave the way in novel avenues of health monitoring systems that can be easily attached to aircraft components, leading to significant weight savings.

## Figures and Tables

**Table 1:** Summary of mechanical properties of multifunctional carbon fiber composites.

Specimen ID	Maximum Compressive Load, P^max^ (N)	Ultimate Compressive Strength, F^cu^ (MPa)
6207-00207	3847	120
6207-00209	4112	127
6207-00210	4370	136
Average	4110	128
Standard Deviation	261.51	8.02
COV	6.36%	6.28%

**Table 2:** Summary of electrical properties of multifunctional carbon fiber composites.

Coupon ID	Maximum Resistance (G Ohms)
6207-00207	42.0
6207-00209	61.20
6207-00210	26.50
Average	43.23
Standard Deviation	17.38

## Data Availability

Not applicable.
